# Microwave Assisted Reactions of Azaheterocycles Formedicinal Chemistry Applications

**DOI:** 10.3390/molecules25030716

**Published:** 2020-02-07

**Authors:** Dorina Amariucai-Mantu, Violeta Mangalagiu, Ramona Danac, Ionel I. Mangalagiu

**Affiliations:** 1 Faculty of Chemistry, Alexandru Ioan Cuza University of Iasi, 11 Carol I, Iasi 700506, Romania; dorina.mantu@uaic.ro (D.A.-M.); rdanac@uaic.ro (R.D.); 2 Institute of Interdisciplinary Research-CERNESIM Center, Alexandru Ioan Cuza University of Iasi, 11 Carol I, Iasi 700506, Romania; violeta.mangalagiu@uaic.ro

**Keywords:** microwave irradiation, five and six members ring azaheterocycles, medicinal chemistry

## Abstract

Microwave (MW) assisted reactions have became a powerful tool in azaheterocycles chemistry during the last decades. Five and six membered ring azaheterocycles are privileged scaffolds in modern medicinal chemistry possessing a large variety of biological activity. This review is focused on the recent relevant advances in the MW assisted reactions applied to azaheterocyclic derivatives and their medicinal chemistry applications from the last five years. The review is divided according to the main series of azaheterocycles, more precisely 5- and 6-membered ring azaheterocycles (with one, two, and more heteroatoms) and their fused analogues. In each case, the reaction pathways, the advantages of using MW, and considerations concerning biological activity of the obtained products were briefly presented.

## 1. Introduction

Over the last three decades, microwave (MW) irradiation has become a successful tool applied in modern medicinal and organic chemistry. This is because MW have several crucial advantages comparative with conventional thermal heating (TH) such us: spectacular accelerations of chemical reactions, shorter reaction times, higher yields and higher product purities. Also, in many cases, under MW, the reactions become eco-friendly (having milder reaction conditions, suppressing side reactions, energy saving, using small amounts, or no organic solvents) [1,2,3,4,5,6,7,8,9,10]. In some cases described in the literature, reactions that did not occur under classical TH were only successful if carried out under MW [1,2,3,4].

The MW effects in chemical reactions rise up intense debates in the scientific community during the time. The effects was explained according with the MW mechanisms of action, while taking into considerations the contribution of electric field component of MW radiation, via three mechanisms: dipolar polarization mechanism, ionic conduction mechanism, and the non-thermal effects of highly polarizing radiation—the so called “specific effects of MW” [5,6,7,8,9,10]. Nowadays, it is almost unanimously accepted by scientists that the effects of MW in chemical reactions it is a complementarity of all of these three mechanisms, in different proportions, according with the environment of chemical reactions.

On the other hand, azaheterocycles are emerging as crucial privileged scaffolds in modern medicinal chemistry and drug discovery, a large variety of biological activities being described for these compounds, such as: antibacterial, antifungal, antitubercular, antiviral, antiplasmodial, antimalarial, anticancer, anti-inflammatory, antihypertensive, diuretics, antithrombics, anticoagulants, antidepressant, anxiolytics, anticonvulsant, analgesic, etc. [11,12,13,14,15].

In this review, we try to present an overview of the newest research in the microwave-assisted reactions applied to azaheterocyclic derivatives and their medicinal chemistry applications, from the literature reported during the last five years.

## 2. Syntheses and Biological Activities of Five- and Six-Membered Ring Azaheterocycles

### 2.1. Five Membered Ring Azaheterocycles

Five membered ring azaheterocycles, especially pyrroles, pyrazoles, imidazoles, triazoles, tetrazoles and their fused derivatives, are multitarget pharmacophores with a large variety of biological activities such as antibacterial, antifungal, antitubercular, antihelmintic, antiplasmodial, anticancer, anti-inflammatory, cardiotonic, antihypertensive, antithrombotic, antihistaminic, antiulcer, analgesic, neuroleptic, etc. [16,17,18,19,20,21,22,23,24]. Having these considerations in view, a lot of attention was paid by researchers for obtaining new compounds with such skeleton, using both conventional TH and MW technology.

#### 2.1.1. Five Membered Ring Azaheterocycles with One and Two Heteroatoms

Alshareef et al. [25] synthesized different pyrrole derivatives, while using the MW assisted Friedländer cyclization method. When substituent Y was a cyano group, pyrrolopyridine derivatives **2a**–**e** were obtained, while when substituent Y was an ester or ketone group, pyrrolopyridine derivatives **2f** and **2g** were obtained. Under MW irradiation, the reactions have some consistent advantages, the reactions time substantially decreased (from hours to minutes) and the yields were 10–20% higher.

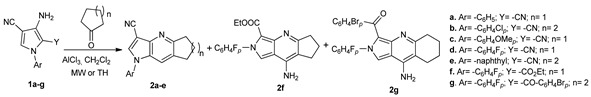



A similar strategy was employed for the synthesis of pyrazolo-pyridine derivatives **4a**–**e** from the corresponding pyrazoles **3**.

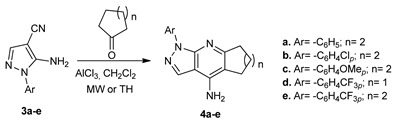



The synthesized compounds were biologically evaluated by Ellman’s test on acetylcholinesterase inhibition, two of them (compounds **2b** (98%) and **4a** (96%)) having a potent inhibition of acetylcholinesterase if compared with the donepezil reference.

In their attempt to synthesize new antiviral compounds, Barradas et al. obtained two new classes of pyrrolo-thiazoles (**7a**–**d** and **10a**–**d**) decorated with a carbohydrate moiety [26]. The syntheses suppose N-alkylations of thiazolederivatives **5** and **8**, while using MW irradiation. It worth to point out that the reactions did not occur under conventional TH conditions.

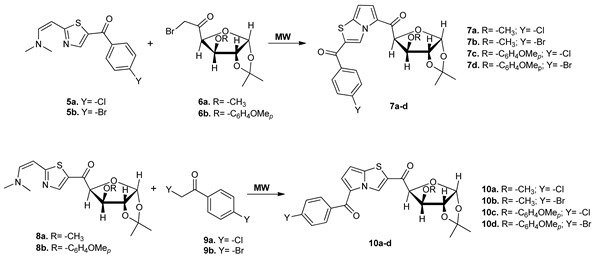



The obtained compounds were tested for their antiviral activity against Junín virus, with all compounds having a very good activity, but, unfortunately and a rather high toxicity.

As part of their research in the thiazoles derivatives series, Cagide et al. [27] synthesized new thiazole-chromene molecules type **13a**,**b** and **14a**,**b**, from 2-amino-thiazole **11a**,**b** and chromone-2-carboxylic acid **12** via an amidation reactions. The synthesis was performed both under conventional TH and MW irradiation, and the comparative study TH versus MW revealed that the yields are roughly the same, but, under MW irradiation, the reactions time substantially decreased and the reagents used are cheaper. Thiazole derivatives have been identified as potent and selective ligands for adenosine receptor.

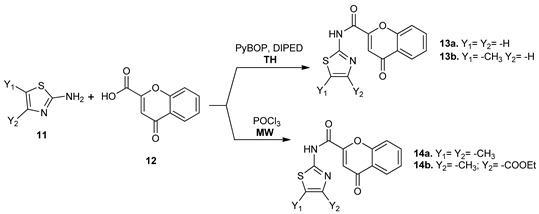



In the pyrazoles derivatives series, Parmar et al. [28] obtained new chromeno-annulated thiopyrano-pyrazoles via an one-pot synthesis type domino-Knoevenagel–hetero-Diels–Alder reaction, using as starting material pyrazolothiones **15a**–**c** and O-alkenyloxy/alkynyloxy-salicylaldehydes/-naphthaldehydes **16a**–**h** and **17a**–**h**, respectively. Under conventional TH conditions, no matter the conditions they employed, no reaction took place. Using microwave irradiation, the reaction successfully occurred in good yields for the desired products (around 70%) and during a short reaction time (12–15 min).

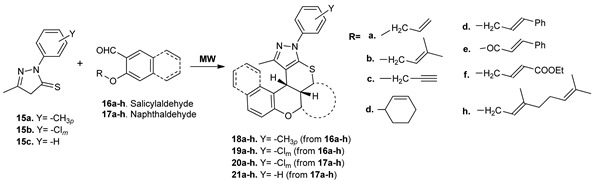



The synthesized compounds were tested for their antibacterial (six strains, Gram positive and Gram negative), antifungal (two strains) and antitubercular (*Mycobacterium Tuberculosis H37RV*) activities. Several compounds (**18a**, **18b**, **18f**, **18h**, **19b**, **19f**, **20a**, **20b**, **21a**) showed good antimicrobial activity (with MIC values in the range of 62.5–25 µg/mL).

Following their work to synthesize new anticancer derivatives, Kovács et al. [29] obtained a new type of steroids bearing an oxa-pyrazole moiety, **24a**–**c** and **25a**–**c**. For this purpose, they used the cyclocondensation reaction of 17β-N-acylhydrazones **22a**–**c** and **23a**–**c**, under both conventional TH and MW irradiation. While using MW irradiation, the reaction took place in a shorter reaction time and the yields of the products were increased.

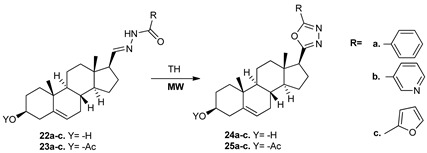



The obtained compounds were tested for their in vitro anticancer activity against four malignant cell lines (HeLa, MCF7, A2780, and A431), and most of them (especially **24** derivatives) exhibited a potent antineoplasic activity against HeLa cells, with one order of magnitude higher that the reference drug, cisplatin.

In the imidazoles series, Saikia et al. [30] synthesized new derivatives with nonsteroidal and steroidal skeleton from the epoxide **26**, by a ring opening reaction while using azaheterocycles. The reaction took place under MW irradiation, the conventional TH being useless for these reactions. In the case of nonsteroidal epoxide **26**, the ring opening of the epoxide is leading to imidazole derivatives **29a**–**c** and **30**.

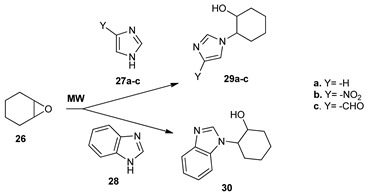



The ring opening of the steroidal epoxide **31** is leading to imidazole derivatives **32a**–**h**.

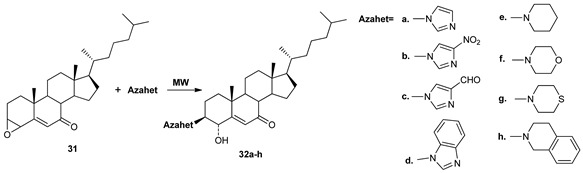



The antimicrobial activities of both imidazole derivatives with nonsteroidal and steroidal skeleton were determined, with compounds **32b** and **32e**–**g** showing a moderate inhibition against bacteria (*Escherichia coli*, *Pseudomonas syringae*, *Bacillus subtilis*, *Proteus vulgaris* and *Staphylococcus aureus*).

While using MW irradiation, Sangi et al. [31] obtained several imidazolidines, oxazolidines, and benzoxazoles. As key intermediate in these reactions, they use 1,1-bis(thiomethyl)-2-nitroethylene **33**, that treated with various alkyldiamines, hydroxylalkylamines, or hydroxylarylamines was leading to the desired compounds (**34**,**35a**–**e**,**36**) in good yields and reduced reaction time (20 min).

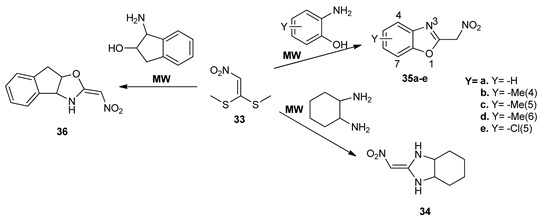



These compounds were tested as acetylcholinesterase (AChE) inhibitors by using an enzyme immobilized capillary reactor-tandem mass spectrometry. The benzoxazole derivative **35d** showed the best inhibition profile.

In their attempt to obtain new antimalarial compounds, Kumar et al. [32] synthesized a series of new benzimidazole (**38a**–**c**) and triazole (**39a**–**o**) derivatives. The synthesis of triazoles derivatives **39a**–**o** took place under MW irradiation (using click chemistry), which allowed for good yields and reduced reaction time (20 min). The obtained benzimidazoles **38a**–**c** and triazoles **39a**–**o** were tested for their antimalarial activity against *Plasmodium falciparum*. Compounds **39a** and **39h** showed the highest antimalarial activity against that parasite, with IC_50_ values in the submicromolar range.

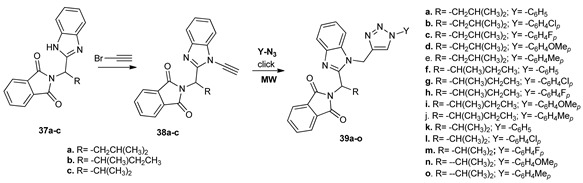



#### 2.1.2. Five Membered Ring Azaheterocycles with More Than Two Heteroatoms

During their research in the triazole series, Srinivas et al. [33] synthesized different 1,2,3-triazole derivatives type **42a**–**g** and **43a**–**g**, bearing a thiazolidinone moiety. The synthesis was achieved by the condensation reaction between 1,2,3-triazoles **41** with a primary aromatic amine and a thioglycolic acid and/orthiomalic acid. The reactions were conducted under conventional TH and MW irradiation, with the use of MW being more reliable: the reaction time substantially decreased (from 2–4 h to 5–10 min) and the yields were higher (with 10–20%).

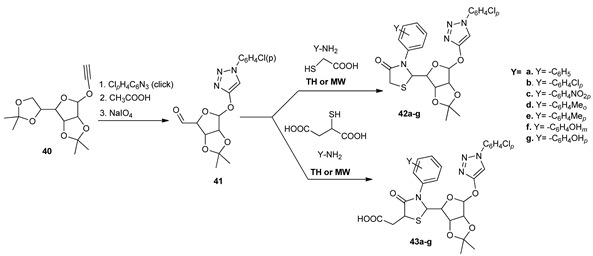



The obtained triazole derivatives **42a**–**g** and **43a**–**g** were evaluated for their anticancer activity on different cancer cell lines, together with a standard drug. Compound **43b** had the best profile in terms of antiproliferative activity (IC_50_ in the micromolar range).

As part of their ongoing work in the azaheterocycles series with antimicrobial activity, Maračić et al. [34]. synthesized three different families of new hybrid 1,2,3-triazole decorated benzo fused heterocycles. For this purpose, they used click chemistry of several azides and the corresponding fused nitrogen heterocycles substituted with a triple bond. Thus, when they used benzoimidazoles and indoles **44a**–**d**, 1,2,3-triazolo-substituted benzimidazole and benzopyrroles **45a**–**s** were obtained, respectively. 1,2,3-Triazolo-substituted benzothiazoles **47a**–**f** were obtained when the benzothiazole **46** was used as propargylated starting compound. The reactions took place both under conventional TH and MW irradiation, the use of MW having the advantages of obtaining slightly higher yields of the products and a substantial decrease of the reaction time from 8 h to 30 min.

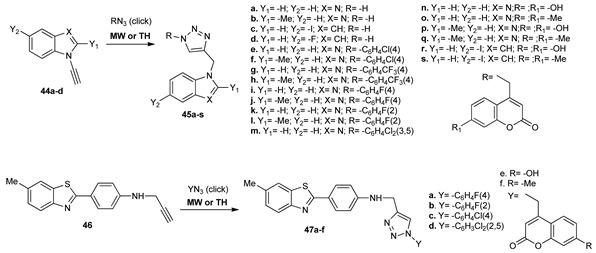



The antibacterial activities against Gram-positive (*Staphylococcus aureus*, *Streptococcus pneumoniae*, *Streptococcus pyogenes*) and Gram-negative (*Escherichia coli*, *Moraxella catarrhalis*, *Haemophilus influenzae*) bacteria have been performed, benzimidazole–1,2,3-triazoles with a p-chlorophenyl and p-fluorophenyl linked to 4-position of the triazole having very good antibacterial activity, with an excellent result against *M. catarrhalis* (MICs 0.5–1 μg/mL).

Using click chemistry (from the corresponding coumarin azide (**48**, **50**) and triple bond compounds), Kraljević et al. [35] obtained 4-substituted 1,2,3-triazole coumarins 49**a**–**p** and 51**a**–**f**. The reaction took place under MW irradiation in good to very good yields (50 to 95%) and one hour reaction time.

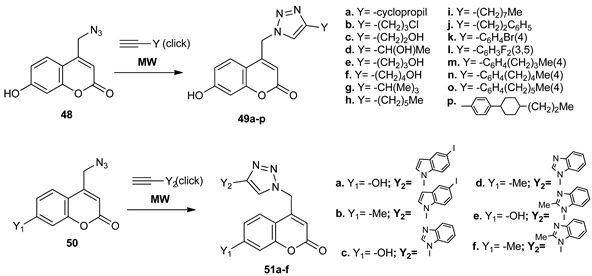



The obtained compounds were tested for their anticancer and antimicrobial activity. The anticancer activity was evaluated in different cancer cell lines, with compounds bearing a 5-iodoindolo moiety (**51a**, **51b**) and benzimidazolo moiety (**51d**, **51f**) having the highest anticancer activity (IC_50_ in the micromolar range). The antimicrobial activity was evaluated against Gram positive and Gram negative strains, three compounds showing high selectivity activity against *Enterococcus* species.

Akhtar et al. [36] prepared a new family of sulfonyl-piperidinyl-1,2,4-triazoles-thioacetamide **54a**–**k** by a relatively facile setup, via thioalkylation of sulfonyl-piperidinyl-1,2,4-triazoles-thiol **52** with activated N-substituted acetamides **53a**–**k**. The reactions were carried out under conventional TH and MW irradiation. Under MW, the setup procedure has some substantial advantages: the yields were higher with 20–40% and the reaction times dramatically decreased from hours (10–20 h) to 30–60 s.

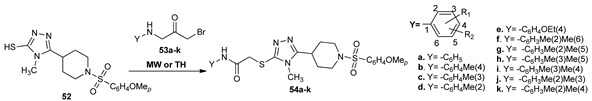



The obtained compounds were tested for their biological activity against carbonic anhydrase (bCA-II), acetylcholinesterase (AChE), and butyrylcholinesterase (BChE) enzymes. Compound **48e** showed very good activity against bCA-II (IC_50_ = 8.69 ± 0.38 µM), AChE (IC_50_ = 11.87 ± 0.19 µM), and BChE (IC_50_ = 26.01 ± 0.55 µM).

Komykhov et al. [37] synthesized a new class of fused 1,2,4-triazolo-pyrimidines **56a**–**h** as part of their effort to obtain new antimicrobial compounds. The reactions were conducted both under conventional TH and MW irradiation, with the use of MW having the advantage of not requiring catalyst.

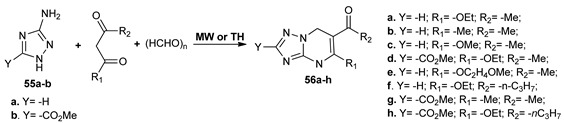



The obtained compounds were tested for their antibacterial and antifungal activity, having a moderate to low antibacterial activity, but significant antifungal activity (MIC between 12–50 µM).

Gomha et al. [38] synthesized a new class of triazolo-, tetrazolo-, imidazolo-, and pyrazolo- fused pyrimidines type **57a**–**f** in their attempt to obtain new antidiabetic compounds.

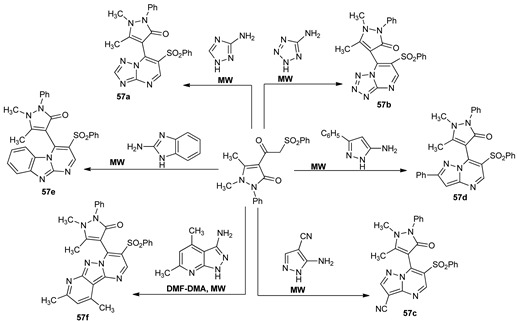



The synthesis was performed in good yields and short reaction time while using MW irradiation via one pot multicomponent reactions from the corresponding pyrazolone and azaheterocycle. The hypoglycemic activity of compounds was evaluated, with compounds **57a** and **57c** having a strong antidiabetic activity with IC_50_ values in nanomolar range.

Chandgude et al. [39] synthesized new tetrazole scaffolds type **58a**–**g** via one pot multicomponent reaction (an amine, a carboxylic acid derivative, an azide, and POCl_3_) while using MW irradiation. The synthesis has the advantages of being stereospecific, in a relatively good yield.

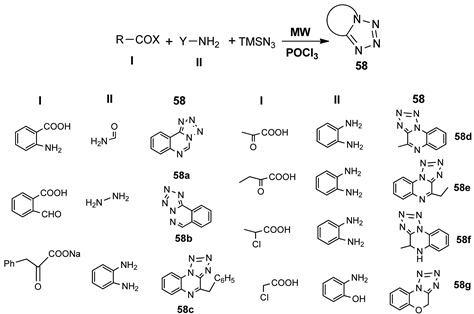



The biological activity (for stroke attack prevention) of compounds was reported to be similar to the one of the marketed drug cilostazol.

### 2.2. Six Membered Ring Azaheterocycles

Six membered ring azaheterocycles are the most significant class of heterocycles used in medicinal chemistry, with these structures playing a key role in current medicinal therapy, many of them already being in use as drugs for humans [11,12]. Compounds containing pyridine, pyridazine, pyrimidine, pyrazine, -and their fused derivatives-, are described to possess a wide range of biological activities, with these including antiviral, anti-HIV, antituberculosis, antimalarial, antileishmanial, antibacterial, antifungal, anti-inflammatory, anticancer, antihypertensive, diuretics, antiaggregative, antiplatelet, cardiotonics, antineurodegenerative, antidepressant, anxiolytics, anticonvulsivant, analgesic, sedatives, antiallergic, etc. [16,21,40,41,42,43,44,45,46,47,48,49,50,51,52,53]. A large variety of syntheses are described for these compounds, with some of them using MW technology.

#### 2.2.1. Six Membered Ring Azaheterocycles with One Heteroatom

While using MW irradiation, Bagley et al. [54] synthesized via cross-coupling Suzuki–Miyaura reactions, three different types of MK2 inhibitors (for treatment of Werner syndrome): quinolines **59a**–**c**, and pyrazoles **60** and **61**.

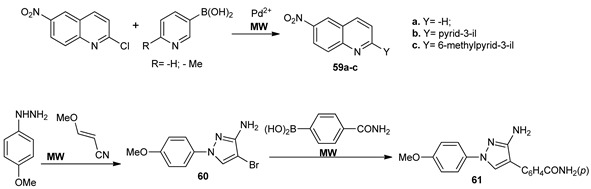



Under conventional TH, the reactions did not take place, while using MW irradiation the desired compounds are obtained in moderate yields. The biological effect of compounds is controversial, with the IC_50_ being roughly at the same level as the lethal dose.

Ajani et al. [55] synthesized a new class of quinolines (**62a**–**l**) bearing hydrazide-hydrazone moiety, while using MW assisted reactions, in continuation of their work in the field of antimicrobial derivatives. The reactions under MW allowed for them to obtain very good yields, high purity of compounds, and a very short reaction time (1–3 min).

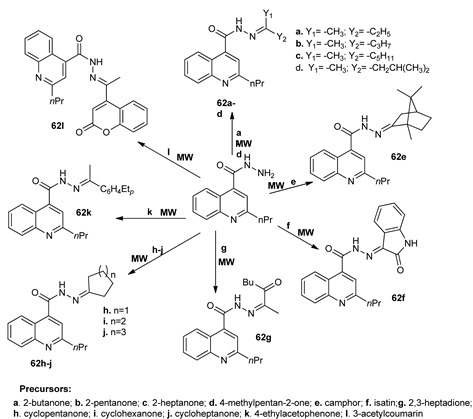



The antimicrobial activity was evaluated against Gram positive and Gram negative strains (six strains), with one compound **62e** having an excellent nonselective antimicrobial activity, with an MIC value in the range of 3.13 to 0.39 μg/mL.

Zahra et al. [56] synthesized a new series of imidazoquinolone esters (**63a**–**f** and **64a**–**d**) and acids (**65a**–**f** and **66a**–**d**), via MW assisted cyclocondensation reactions of 7,8-diaminoquinolone and arene/heteroarenecarboxaldehydes. The uses of MW allow for excellent yields, high purity of compounds, and a short reaction time (8–10 min).

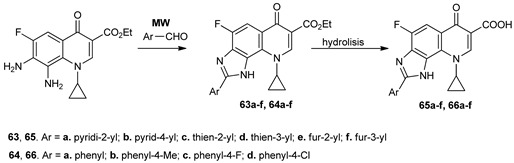



The antimicrobial activity of imidazo-quinolone esters and acids were evaluated against bacteria (Gram positive and Gram negative) and fungi, with both classes of compounds having good antibacterial activity. Among the tested compounds, compounds **65e**,**f** and **66c**,**d** have excellent activity against two Gram-positive strains, *Bacillus subtilis* and *Haemophilus influenzae*.

Thangaraj et al. [57] synthesized a new series of quinolinederivatives **67a**–**h** under MW irradiation while using an Ugi one pot four component condensation of lipoic acid, cyclohexylisocyanide, aniline derivatives and 2-methoxy quinoline-3-carbaldehyde derivatives. The use of MW has the advantages of excellent yields, high purity of compounds, the use of inexpensive solvents, and a short reaction time (15 min).

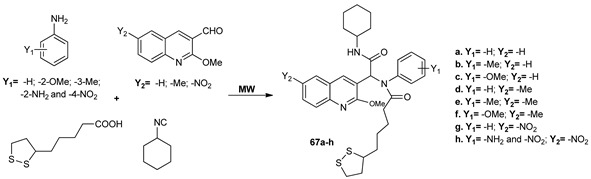



The synthesized compounds were evaluated for the in vitro antibacterial and antioxidant potency. The antimicrobial assay revealed that four quinolines (**67b**, **67c**, **67g**, and **67h**) have a very good antimicrobialactivity against *Bacillus cereus*, *Staphylococcus aureus*, *Escherichia coli*, *Enterococcus faecalis Candidaalbicans* and *Candida utilis*. The study regarding the antioxidant properties revealed that three quinoline peptides (**67b**, **67g** and **67h**) are effective on DPPH scavenging agents.

Subashini et al. [58] synthesized new quinolyl-quinolinones compounds **68a**–**h** via a Friedlander reaction, under conventional TH and MW irradiation. Under MW irradiation, the reaction setups have the advantages of higher yields and purities of compounds, as well as shorter reaction time (from 7–9 h to 4–7 min).

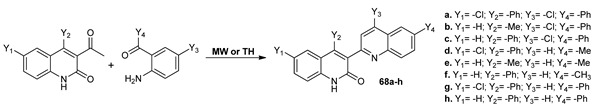



The obtained compounds were the subject of antimicrobial and antioxidant assay. The results showed that all of the compounds possess antibacterial activity (in good to moderate range) and are also potent as antioxidants (comparable to the standard ascorbic acid).

Mokaber–Esfahani et al. [59] obtained new pyrimidyl-quinolinones derivatives **69a**–**g** under conventional TH and MW irradiation. By comparison with conventional TH, the MW irradiation has the advantages of higher yields and shorter reaction time (from 4–8 h to 15–30 min).

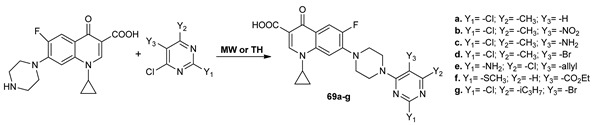



The antimicrobial activity was evaluated against Gram positive and Gram negative strains, with two compounds **69a** and **69c** having excellent antimicrobial activity, superior to reference drug ciprofloxacin.

Using MW irradiation and conventional TH, Shekarrao et al. [60] synthesized new fused pyridines **70a**–**g**, quinolines **71a**–**c**, pyrimidines **72a**–**h**, and quinazolines **73a**–**c**, with pyrazole skeleton. Under MW irradiation, the yields are higher and the reaction times decrease substantially (from 24 h to 15 min).

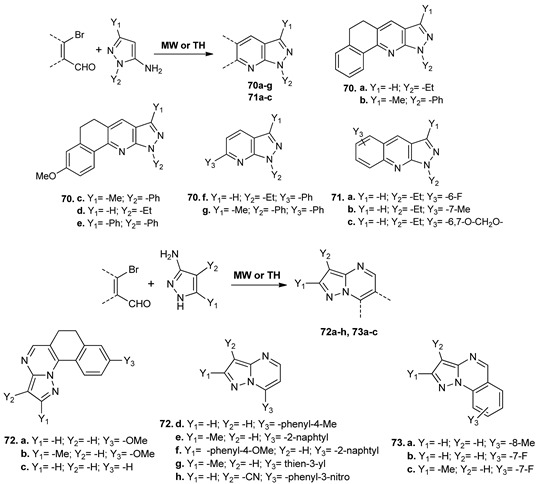



Four of the synthesized fused azaheterocycles (pyridine **70a**, quinoline **71c**, pyrimidines **72a** and **72d**) showed excellent anticancer activity, which was comparable to reference drug doxorubicin.

In continuation of their seeking for new anticancer compounds, Cortés et al. [61] synthesized isoquinoline based derivatives **74a**–**c**, by conventional TH and MW irradiation, the last one having the advantages of higher yields, milder conditions, and shorter reaction time.

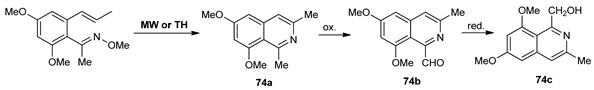



The in vitro anticancer assay revealed that the isoquinoline derivatives **74a**–**c** possess very good activity against lung cancer and two breast cancer cell lines (IC_50_ values in the micromolar range).

De Nisco et al. [62] synthesized a series of pyridophenoxazinone isomers **75a**–**f** and 75*a–**f** with antiproliferative activity, while using quinoline-5,8-dione and 2-aminophenols as starting materials. Under conventional TH, they only obtained trace of desired compounds **75a**–**f** and 75*a–**f**, while the MW assisted reactions lead to the desired products in good yields.

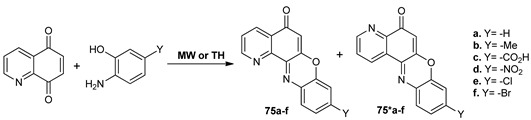



The pyridophenoxazinone isomers **75a**–**f** and 75*a–**f** were found to exhibit good anticancer activity.

#### 2.2.2. Six Membered Ring Azaheterocycles with Two and More Heteroatoms

Mantu et al. [63] developed a convenient, simple, and efficient synthesis of fused tetra- and penta-cyclic 1,2-diazines using MW and ultrasound (US) irradiation in continuation of their research in the field of biologically active compounds with 1,2-diazine skeleton. In this respect, they synthesized two new classes of fused pyridazine, tetracyclic benzoisoindolo-pyridazine **78a**–**c** and pentacyclic benzoisoindolo-phthalazine **79a**–**c**, from the corresponding pyridazine and phthalazine salts (**76a**–**c**, **77a**–**c**) and 1,4-naphthoquinone, via a Huisgen 3 + 2 dipolar cycloaddition reaction. If compared with conventional TH, under MW and US irradiation, the products yields were higher (in some cases almost double), the reaction time is considerably reduced (from hours to minutes) and the consumed energy substantially decreased.

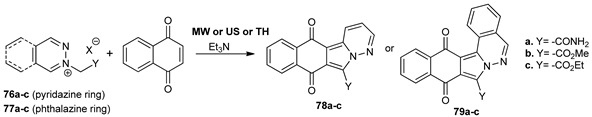



The penta- and tetra-cyclic 1,2-diazines were evaluated for their in vitro anticancer activity. The pentacyclic 1,2-diazine derivatives exhibit significant anticancer activity against Breast Cancer MCF7, Leukemia MOLT-4, Leukemia CCRF-CEM, and Non-Small Cell Lung Cancer NCIH460. A feasible mechanisms of action for anticancer efficiency of the pentacyclic 1,2-diazines **79a**–**c** have been furnished.

In a comprehensive work, Zbancioc et al. [64] performed a thorough study concerning the preparation of 1,2-diazine with dihydroxyacetophenone skeleton and their antimicrobial and antitumoral activity. The synthesis of the desired pyridazine **81a**–**e** and phthalazine **82a**–**e** derivatives was performed under conventional TH and while using MW and US irradiation, by direct alkylation of nitrogen heterocycles with α-bromodihydroxyacetophenones **80a**–**e**. Under MW irradiation, the reaction time dramatically decreases (from 48 h to 5 min) and, in some cases, the yields were higher.

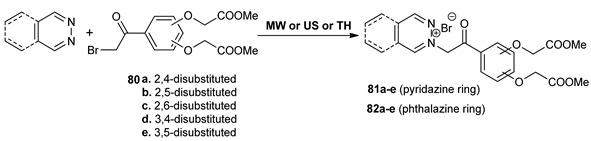



The in vitro antimicrobial and anticancer assay reveals that compounds have a good potency in terms of antibacterial activity (against Gram-positive and Gram-negative strains) and moderate antifungal and anticancer activity (against HeLa cells).

Rehman et al. [65] synthesized new pyrimidines (**83a**–**m**) that were decorated with pyridine and sulfonamide moieties of potential interest in Alzheimer’s disease. The syntheses were carried out under conventional TH and MW irradiation. Under MW irradiation, the reaction time is reduced about five folds and the yields were slightly higher.

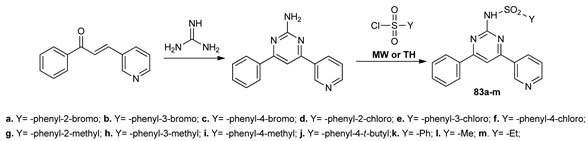



The synthesized compounds were evaluated against acetylcholinesterase (AChE) and butyrylcholinesterase (BChE), because these enzymes play a crucial role in the treatment of Alzheimer’s disease. Compounds **83i** and **83l** were found to be the most active among this series (IC_50_ in the range of 3–5 μM).

Hosamani et al. [66] developed a simple and convenient method for synthesis of pyrimidine bearing a chromen-2-one moiety, under conventional TH and MW irradiation. By comparison with reaction under classical TH, under MW irradiation the yields were higher (with about 20–25%) and the reaction time substantially decreased, from about 800 to 15 min.

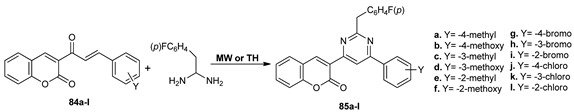



The in vitro anticancer assay (on human lung carcinomaA-549 and human adenocarcinoma mammary glandMDA-MB-231) reveals that the obtained compounds have significant anticancer activity, with two of compounds **83b** and **83j** being superior to the standard drug Cisplatin.

Kaoukabi et al. [67] synthesized two new classes of pyrimidine derivatives **86a**–**f** and **87a**–**l** with antiviral activity, while using both conventional TH and MW irradiation. The advantages of using MW are better yields (increased with about 50%) and smaller reaction times (times decreased from hours to minutes).

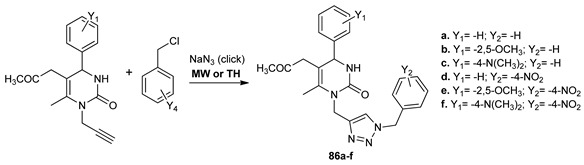

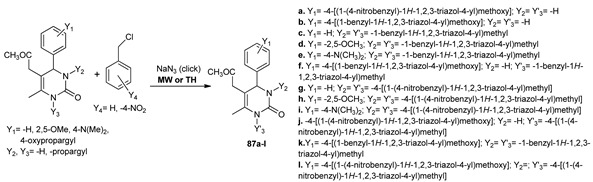





The obtained compounds were evaluated for their antiviral activity, with four of the tested compounds (**87c**, **d**, **f**, **g**) having a substantial activity, with an EC_50_ in the range of 3–10 μM.

Khaldi-Khellafi et al. [68] synthesized new pyrimidine derivatives **88a**–**n** analogs of curcumin in their attempt to obtain compounds with antioxidant and antibacterial activity. The synthesis was performed by MW irradiation and conventional TH. Under MW irradiation, the reaction time decreased substantially from 6–9 h to 3–5 min and the yields were roughly the same in both methods.

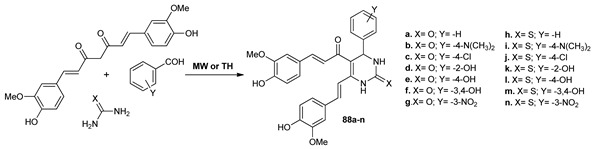



The biological activity data of the synthesized compounds showed that most of them exhibited greater antioxidant and antibacterial activity than the standard drug curcumin.

Bhatia et al. [69] synthesized a new class of bis-pyrimidine derivatives **89a**–**j** with antibacterial activity. The synthesis was performed by MW irradiation and conventional TH. Under MW irradiation, the yields were higher, with about 10–15%, and the reaction time decreased substantially from 6–15 h to 10–15 min.

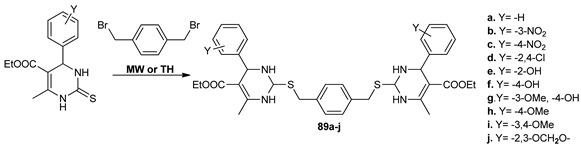



The obtained compounds were screened for their antimicrobial activity against Gram-positive and Gram-negative strains and several fungi. The obtained results reveal that compounds with electron-donating substituent (Y) on the phenyl ring have remarkable antibacterial activity, while compounds having electron-withdrawing substituent exhibited excellent antifungal activity.

In a subsequent paper, Bhatia et al. [70] synthesized another series of bis-pyrimidine derivatives **90a**–**j** with antibacterial activity. The synthesis was performed again by MW irradiation and conventional TH, under MW irradiation the yields being higher, the reaction time decreased and the work-up conditions were milder.

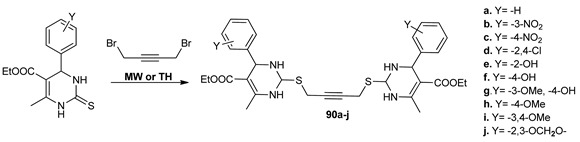



The in vitro antimicrobial assay (Gram-positive and Gram-negative strains, fungi) revealed that compounds **90** showed good antibacterial and antifungal activities. However, compound **90j** was the most active in terms of antibacterial potency, while compound **90b** was the most potent in terms of antifungal properties.

Sompalle et al. [71] synthesized a new class of fused triazoloquinazoline derivatives **91a**–**o**, while using MW irradiation and conventional TH. No matter the conditions were employed, under conventional TH, the reactions did not take place, while, under MW irradiation, the compounds were obtained in good yields and short period of time (~3 min). The compounds were tested for their antioxidant activity, all presenting a moderate activity.

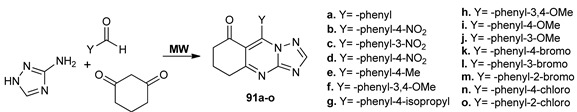



Following their goal to obtain new adenosine receptor (AR) antagonists, Burbiel et al. [72] synthesized several classes of triazoloquinazolines **94**–**95**. In this respect, in the first step, 2-aminobenzonitrile was treated with the appropriate acyl halides and then in the second step with hydrazine hydrate, when quinazoline derivatives **92a**–**m** were obtained. The reactions were performed under both MW irradiation and conventional TH. In the case of aliphatic acyl halides, the un-cyclized intermediates **93a**–**m** were obtained, but the separation of this intermediate was not necessary, because both of the isomers lead to the same triazoloquinazolines **94a**–**m** and **95a**–**c** derivatives in the subsequent condensation step with all different employed reagents. Under MW irradiation, the reaction time substantially decreased from 5–24 h to 10–20 min and the yields were slightly higher.

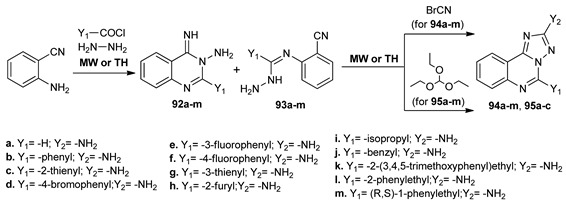



The affinity of obtained compounds as AR antagonists was tested. In many cases, significantly different affinities for human and rat receptors were observed, with low nanomolar Ki values.

## 3. Concluding Remarks

No doubt, by comparison with conventional TH, microwave assisted reactions offer several crucial advantages, such as spectacular accelerations of chemical reactions, shorter reaction times, higher yields and higher product purities, eco-friendly synthesis, and reactions that only occur under MW irradiation. As a result, MW assisted reactions remain a useful and an alternative tool for conventional TH in modern medicinal chemistry.
